# Sparse Coding Models Can Exhibit Decreasing Sparseness while Learning Sparse Codes for Natural Images

**DOI:** 10.1371/journal.pcbi.1003182

**Published:** 2013-08-29

**Authors:** Joel Zylberberg, Michael Robert DeWeese

**Affiliations:** 1 Department of Physics, University of California, Berkeley, Berkeley, California, United States of America; 2 Redwood Center for Theoretical Neuroscience, University of California, Berkeley, Berkeley, California, United States of America; 3 Helen Wills Neuroscience Institute, University of California, Berkeley, Berkeley, California, United States of America; Indiana University, United States of America

## Abstract

The sparse coding hypothesis has enjoyed much success in predicting response properties of simple cells in primary visual cortex (V1) based solely on the statistics of natural scenes. In typical sparse coding models, model neuron activities and receptive fields are optimized to accurately represent input stimuli using the least amount of neural activity. As these networks develop to represent a given class of stimulus, the receptive fields are refined so that they capture the most important stimulus features. Intuitively, this is expected to result in sparser network activity over time. Recent experiments, however, show that stimulus-evoked activity in ferret V1 becomes *less* sparse during development, presenting an apparent challenge to the sparse coding hypothesis. Here we demonstrate that some sparse coding models, such as those employing homeostatic mechanisms on neural firing rates, can exhibit decreasing sparseness during learning, while still achieving good agreement with mature V1 receptive field shapes and a reasonably sparse mature network state. We conclude that observed developmental trends do not rule out sparseness as a principle of neural coding *per se*: a mature network can perform sparse coding even if sparseness decreases somewhat during development. To make comparisons between model and physiological receptive fields, we introduce a new nonparametric method for comparing receptive field shapes using image registration techniques.

## Author Summary

The popular sparse coding theory posits that the receptive fields of visual cortical neurons maximize the efficiency of the neural representation of natural images. Models implementing this idea typically minimize a combination of the error in reconstructing natural images from neural activities, and the average level of activity in the model neurons. In simulations, these models are presented with natural images and the RFs then develop so as to increase representation efficiency. After a long developmental period, the model RFs typically agree well with those observed experimentally in visual cortex. Since the models seek to minimize (for a given level of reconstruction error) the neural activity levels, the average levels of neural activity might be expected to decrease as the models develop. In the developing mammalian cortex, visual RFs are also modified during development, so the sparse coding hypothesis might appear to suggest that activity levels should decrease during development. Recent experiments with young ferrets show the opposite trend: mature animals tend to have more active visual cortices. Herein, we demonstrate that, depending on the models' initial conditions, some sparse coding models can exhibit increasing activity levels while learning the same types of RFs that are observed in visual cortex: the developmental data do not preclude sparse coding.

## Introduction

A central question in systems neuroscience is whether optimization principles can account for the architecture and physiology of the nervous system. One candidate principle is sparse coding (SC), which posits that neurons encode input stimuli *efficiently*: stimuli should be encoded with maximum fidelity while simultaneously using the smallest possible amount of neural activity [1], [2]. Much evidence suggests that primary visual cortex (V1) forms sparse representations of visual stimuli [1], [3]–[7]. For example, when trained with natural scenes, SC models have been shown to learn the same types of receptive fields (RFs) as are exhibited by simple cells in macaque primary visual cortex (V1) [1], [8].

Throughout this paper, we make reference to the notion of “sparseness”. Intuitively, sparseness is related to there being either a small subset of neurons active at any time (population sparseness), or to each neuron being active only a small fraction of the time (lifetime sparseness) [9]. In the Methods section, we define the precise notions of sparseness that we use in this paper.

In further support of the SC hypothesis, measurements of the firing rates of V1 neurons in response to videos of natural scenes show that those rates are low, and that the firing rate distributions are sharply peaked near zero [7]. Similarly, cell-attached recordings in auditory cortex show highly sparse levels of activity [10]. Conversely, other experimenters [11] have observed non-sparse (dense) neuronal activity in visual cortex, although the boundary between “sparse” and “dense” activity is open to interpretation and thus it is unclear how sparse the activity must be in order to confirm the SC hypothesis [12]. Importantly, however, it has been observed that stimulating larger portions of the visual field leads to sparser, and less correlated, V1 neuronal responses [4]–[6]. It has been suggested that this effect arises because of inhibitory recurrent connections between excitatory cells, mediated by the appropriate interneurons [6].

**Figure 1:**
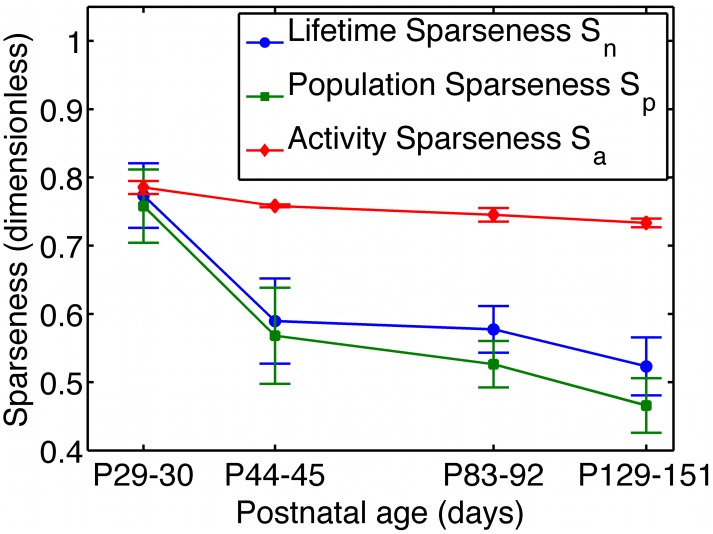
V1 developmental data appear to challenge the canonical sparse coding models. Multi-unit activity in primary visual cortex (V1) of awake young ferrets watching natural movies shows decreasing sparseness over time. The sparseness metrics shown in this figure are defined in the results section of this paper, and the data are courtesy of Pietro Berkes [14], [15]. The plot has a logarithmic horizontal axis. For contrast, one expects that, in sparse coding models, the sparseness should increase over time. This point was emphasized in recent work [14]. In this paper, we show that, in sparse coding models sparseness can actually decrease during the learning process, so the data shown here cannot rule out sparse coding as a theory of sensory coding.

In simulating the development of a sparse coding model, one typically [1], [3] initializes the receptive fields with random white noise — so as to not bias the shapes of the RFs learned by the network — and then presents the network with natural images, in response to which the RFs get modified. As the model (*e.g.*, [1], [3], [13]) modifies itself in response to the stimuli, neurons gradually learn features that allow for a better encoding of the stimuli, so the sparseness is expected to increase over time. This point was emphasized in recent work [14]. Physiology experiments, however, show something different in the developing visual cortex. Recently, Berkes and colleagues measured multi-unit V1 activity in awake young ferrets viewing natural movies, and found that, as the animals matured, their stimulus-driven V1 activity became *less* sparse [14], [15] (Fig. 1).

The above discussion hints at a major source of confusion in this area of research. In particular, sparseness is discussed as both a relative measure (*i.e.*: “Is network A sparser than network B?”), and as an absolute descriptor (*i.e.*: “Is network A sparse?”). In this paper, we will first study relative measures of sparseness, and observe how these measures change as a result of development in our recently published SAILnet model [12]; do they increase, or decrease over time? The absolute sparseness values of the final (mature) networks – which vary between 0 (not sparse), and 1 (maximally sparse) – will be used to infer whether the final network is sparse at all. This mirrors the way that Berkes and colleagues discussed sparseness in the developing ferret.

The ferret sparseness-over-time data appears to contradict the SC hypothesis. At the same time, that hypothesis has otherwise been quite successful in explaining some key features of peripheral sensory systems. It is therefore natural to ask whether sparse coding models necessarily *must* exhibit increasing sparseness in order to learn V1-like receptive fields and perform sparse coding in the mature state. In this work, we focus primarily on a recently published variant of sparse coding called SAILnet [12] in which homeostasis regulates the neuronal firing rates while synaptically local plasticity rules modify the network structure, leading to V1-like receptive field formation. We will demonstrate that, depending on the initial conditions of the simulation, SAILnet can exhibit either increasing, or decreasing sparseness, while learning RFs that are in quantitatively good agreement with those observed in V1, and having a reasonably sparse final state. The choices of parameter values in the model determine the equilibrium state to which the network ultimately converges. If the initial conditions are *even sparser* than this equilibrium point, sparseness will decrease during development, and yet the final state can still be sparse in an absolute sense.

We will also see that, for appropriately chosen initial conditions, the same can be true of the canonical SparseNet model of Olshausen and Field [3]. Thus, the apparent contradiction between the ferret developmental sparseness data, and SC models [14] does not necessarily mean that SC is implausible as a theory for sensory computation. Later in this paper, we discuss plausible alternatives for sensory coding other than SC models.

## Results

### Overview of the Sparse and Independent Local network (SAILnet) model

**Figure 2:**
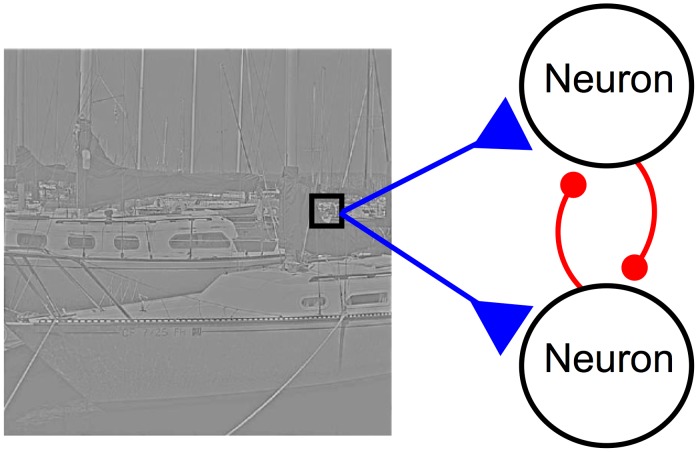
SAILnet architecture. In our model, described in detail elsewhere [12], leaky integrate-and-fire neurons receive inputs from pixels in whitened natural images, in a rough approximation of the thalamic input to V1. Inhibitory recurrent connections between neurons, shown in red, act to decorrelate the neuronal activities. The neurons have variable firing thresholds, which are varied by the neurons so as to maintain a desired long-term-average firing rate.

Since this paper focuses primarily on our SAILnet model (Fig. 2), we will now provide a brief overview that model, which is described in detail elsewhere [12] and summarized in the Methods section. The model consists of a network of leaky integrate-and-fire (LIF) neurons, which receive feed-forward input from image pixels, in a rough approximation of the thalamic input to V1. The neurons inhibit each other via recurrent inhibitory connections, the strengths of which are learned so as to reduce correlations amongst the units, consistent with recent physiology experiments [4]–[6]. We note that one can modify SAILnet so that interneurons mediate the inhibition between excitatory cells so as to satisfy Dale's law (E-I Net; [16]).

The neurons' firing thresholds are modified over time so as to maintain a target lifetime-average firing rate. For our LIF neurons, this is similar to synaptic rescaling, which has been proposed as a mechanism to stabilize correlation-based learning schemes [17], [18], and has been observed in physiology experiments [17]. Alternatively, the variable firing threshold can be thought of in terms of a modifiable intrinsic neuronal excitability, another well-known homeostatic mechanism [19].

Finally, the feed-forward weights are learned by the network, so that the neuronal activities form an optimal linear generative model of the input stimulus, subject to the constraints imposed by limited firing rates and minimal correlations. The derivation of our learning rules from this objective function is presented in [12]. All information needed for the model's plasticity rules is available locally at the synapse being modified — updates depend only on the pre- and post-synaptic activity levels.

### SAILnet activity can become less sparse during receptive field formation, much like ferret V1 development

To study the change in sparseness over time, we ran SAILnet simulations, starting with randomized feed-forward weights, recurrent connection strengths, and firing thresholds that were initialized with Gaussian-distributed white noise. At different times during the development process, we recorded the simulated neuronal activity in response to randomly selected batches of natural images. Following a recent experimental study [14], we computed from these network activities three sparseness measures, which are discussed in more detail in the Methods section. Each of these measures varies between 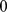 (not sparse at all) and 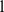 (as sparse as possible).

The first of these, the “activity sparseness,” 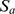, measures the fraction of units that are *inactive* in response to a given stimulus, averaged over different stimuli. If this quantity is near 1, then only a small subset of units responds to each stimulus. If every unit is active in response to every stimulus, then 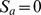.

The “population sparseness” [5], [14], [20], 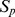, measures the degree to which the population response to a given stimulus is restricted to a small subset of the population, averaged over all stimuli. If only a small number of units have large activities in response to a given stimulus, then 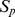 will be near 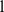, even if many units have small but non-zero activities. By contrast, 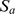 would be small in that case, because there are not many completely inactive units. If the units all respond equally to every stimulus, then 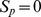. For the same level of representation error, the formation of efficient representations demands relatively high values of 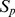 and 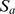, such that only a small fraction of the available neural resources are utilized in representing each image.

Finally, the “lifetime sparseness” [5], [14], [20] measures how much the responses of individual units tend to be concentrated over a small subset of stimuli, averaged over units. If the units respond very selectively, so that they have strong responses to a small number of stimuli, and weak responses to most stimuli, then 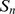 will be near 1. Conversely, if each unit responds equally to all stimuli, then 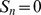.

Intuitively, all of these measures are somewhat related (although, see [9] for notable exceptions). At the same time, when it comes to the efficiency of the neural representation, the more relevant quantities are the activity sparseness and the population sparseness, which both have to do with the fraction of neural resources used to represent each image [9]. Furthermore, the values of “lifetime” sparseness one obtains will vary with the time scale over which one performs the measurement. As such, it is a somewhat more ambiguous quantity than are the activity and population sparseness measures. Despite these issues, we include results for the lifetime sparseness for our model in keeping with the experimental study [14] that motivated this theoretical project.

**Figure 3:**
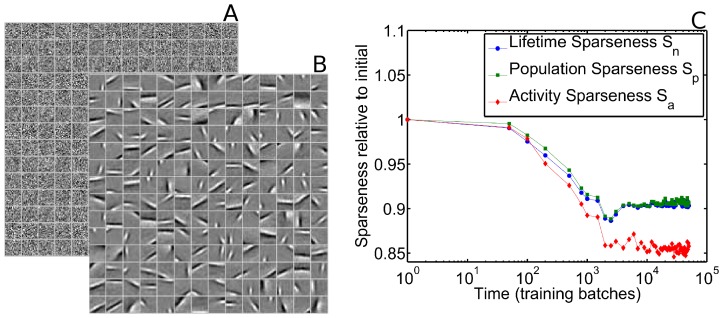
SAILnet multi-unit activity can become less sparse during receptive field formation. A SAILnet simulation was performed in which the RFs, firing thresholds, and recurrent connection strengths were initialized with random numbers (see Methods section for details). (**A**) These initial RFs are shown for 196 randomly selected model neurons. Each box on the grid shows the RF of one neuron, with white corresponding to positive pixel values, and black corresponding to negative ones. (**B**) After training with natural images, these same SAILnet neurons have oriented, localized RFs. (**C**) All three of our “multi-unit” sparseness measures decrease during the training period, as has been observed in the visual cortex of maturing ferrets [14]. We made similar observations when we made measurements of single-neuron sparseness values (data not shown).

In order to further facilitate meaningful comparison with the experiment of Berkes and colleagues, we mimicked a multi-unit activity measurement by randomly grouping together sets of 8 SAILnet neurons, whose activities were then summed to form a multi-unit response. These “multi-unit” activities were used for computing our sparseness measures. This procedure yielded results (Fig. 3) that were qualitatively similar to the single-unit sparseness measures (not shown), but with larger changes in sparseness values. A direct quantitative comparison between our model multi-unit sparseness data and the ferret data is difficult because it is not clear how best to estimate the relevant number of neurons to group together, or even whether all groupings should have the same number of neurons. Furthermore, in the ferret data, the neurons grouped together are physically nearby, which means that, due to retinotopic and orientation maps in V1, they will have similar receptive fields. The SAILnet model has no such notion of spatial organization and our random grouping of cells misses that aspect of the ferret experiment.

In Fig. 3, we show a random subset of 196 (out of the 250 total) receptive fields of SAILnet neurons both before and after the network is trained with natural scenes. We also show the evolution of our multi-unit sparseness measures during that training process. Contrary to the idea that sparse coding models must show strictly increasing sparseness during learning [14], our SAILnet model can display *decreasing* sparseness by all three measures while it is learning localized and oriented receptive fields. We will later show that the popular SparseNet model of Olshausen and Field [3] can also exhibit decreasing sparseness over time.

The time course of the sparseness measures depends on the learning rates (parameter modification step sizes), with smaller learning rates leading to slower changes in sparseness measures, as expected (data not shown). The depth of the observed “undershoot” also depends on the initial conditions and the learning rates. The specific activity sparseness values (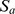) depend on the chosen threshold: higher thresholds lead to higher sparseness values.

The receptive fields learned by the model, while displaying decreasing sparseness, are in good quantitative agreement with a measured corpus of 250 macaque monkey V1 simple cell receptive fields, as we will demonstrate in the section on comparisons of receptive field shapes.

The model discussed in this section and shown in Fig. 3 has relatively high firing thresholds and a relatively large amount of lateral inhibition in the initial state. Those properties lead to highly sparse firing. As the network learns, the homeostatic firing rate regulation reduces the thresholds and then inhibitory connections are modified by their own plasticity rules (see Methods section for details). These have the effect of reducing the model's sparseness over time. For contrast, in the next section we will consider a model that is identical to the one presented here, but with the following modifications to the initial conditions: the firing thresholds are initialized at smaller values and there is initially less lateral inhibition.

### For less sparse initial conditions, SAILnet multi-unit activity becomes sparser during receptive field formation

We find that SAILnet does not *require* sparseness to decrease over time; rather, it is *compatible* with decreasing sparseness. To demonstrate this point, we repeated our SAILnet simulations and sparseness measurements with different initial conditions (see Methods section for details). As discussed in the previous section, these initial conditions have lower firing thresholds and less lateral inhibition than in the model shown in Fig. 3.

**Figure 4:**
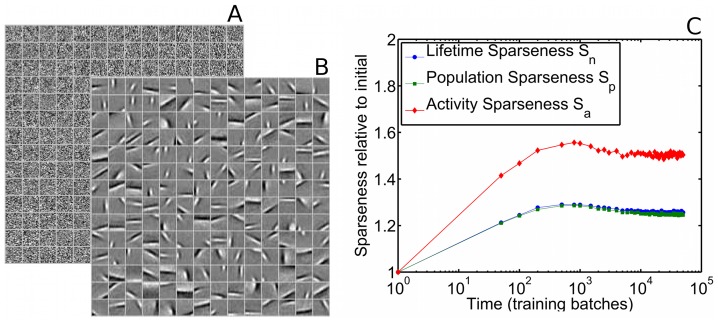
For less sparse initial conditions, SAILnet multi-unit sparseness measures increase during training. A SAILnet simulation was performed in which the RFs were initially randomized, and the recurrent inhibitory connection strengths and firing thresholds were initialized with random numbers that were smaller than for the simulation described in Fig. 3 (see Methods section for details). (**A**) The initial RFs are shown for 196 randomly selected model neurons. As in Fig. 3, each box on the grid depicts the RF of one neuron, with lighter tones corresponding to positive pixel values, and darker tones corresponding to negative values. (**B**) After training with natural images, these same SAILnet neurons have oriented, localized RFs. (**C**) All three of our multi-unit sparseness measures increase during the training period. Aside from the initial conditions, the network used to generate these data was identical to the one from Fig. 3: both networks have the same learning rates, the same number of neurons, the same target mean firing rate, and are trained on the same database of whitened natural images.

In this case, the relatively low firing thresholds and relatively small amount of lateral inhibition lead to the initial network state being less sparse than the final (equilibrium) state, so sparseness increases over time (Fig. 4).

Similar to Fig. 3, in Fig. 4 we show a random subset of 196 neuronal receptive fields both before and after the training procedure and, as we demonstrate below, those RF shapes are in quantitative agreement with those measured in macaque V1.

We emphasize that, compared to the model discussed in Fig. 3, which exhibited increasing sparseness, the model discussed here (and in Fig. 4) differs only in the initial conditions; all other parameters were the same for the two models. Consequently, after a long training period, over which the effects of the initial conditions gradually disappear, these two models have very similar final sparseness levels and receptive fields. For the models studied in this paper (Figs. 3 and 4), the final multi-unit sparseness values (after the final training batch) are 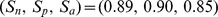 for the model in which sparseness increases over time (Fig. 4), and 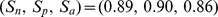 for the model in which sparseness decreases over time.

### Theoretical models besides SAILnet can also display both increasing and decreasing sparseness over time

While our SAILnet model [12] and a recent extension that obeys Dale's law [16] are more biophysically realistic than previous sparse coding models, the increasing or decreasing sparseness over time we describe above (Figs. 3 and 4) is not unique to SAILnet.

To explore this issue more fully, we return to the canonical SparseNet model of Olshausen and Field [3], [21]. We first note that, as in SAILnet, the “equilibrium” sparseness level in SparseNet is determined by a free parameter in the model (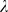 in [3]). Furthermore, in the SparseNet model, there is a homeostatic mechanism that adjusts the magnitudes of the feed-forward weights so as to keep the units' activities near some pre-defined set point. Similar to SAILnet, this process is not instantaneous.

In what follows, we use the SparseNet code of Olshausen and Field [3], [21] “out of the box,” without modifying any parameters except the initialization of the basis functions — these are analogous to the feed-forward weights, or receptive fields, of SAILnet units.

**Figure 5:**
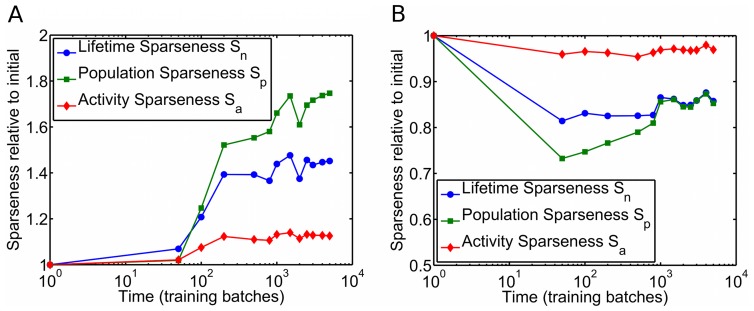
SparseNet can also display either increasing or decreasing sparseness during learning. To check that our conclusions apply to other models besides SAILnet, we performed simulations with the publicly available SparseNet code of Olshausen and Field [3], [21]. (**A**) When the basis functions are initialized with large-amplitude white noise (see text for details), the sparseness increases over time contrary to the ferret data shown in Fig. 1. (**B**) However, when the bases are initialized with small-amplitude white noise, the sparseness decreases over time.

We begin by initializing these basis functions with Gaussian white noise of variance 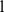, so that the 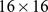 bases have 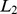 norms of approximately 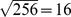. In this case, sparseness increases over time (Fig. 5a) and the basis amplitudes decrease: the mean 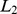 norm of these bases is approximately 0.5 once the model converges, after the training period.

These changes can be understood by recalling that, during inference — where the activities of the units are determined in response to a given image — the activities are chosen to minimize the following cost function: 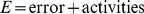. The error, as in SAILnet, is the sum over pixels of the squared error of the difference between the image and a linear generative model formed by multiplying each unit's activity by its basis function. Thus, if the basis has a small magnitude, then large activations are needed in order to form a decent linear generative model. However, these large activations are punished by the “activities” term in the cost function. As a result, some of the units that add only a modest improvement to the representation are turned off, or nearly so. If the basis has a large magnitude, then only small activations are needed to form the linear generative model, and these small activations are less strongly penalized than are large activations. As a result, the activation can be more distributed over the network when the filters have large magnitudes. To summarize: large basis function magnitudes lead to less sparse network activity and the basis magnitudes are modified with a non-zero timescale.

Putting all of this together, if we initialize the bases with Gaussian white noise of variance 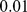, so that they initially have 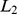 norms in the neighborhood of 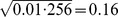, then the basis norms increase over time during training. After this model converges, the mean 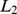 norm of the bases is again around 0.5. Consequently, the sparseness decreases over time (Fig. 5b).

As in SAILnet, one way to understand these trends is to recall that the model parameters dictate the final “equilibrium” state of the model, but the initial conditions can be chosen independently of the final state. As such, initial conditions can be chosen to be either more or less sparse than the equilibrium condition, leading to sparseness either decreasing or increasing over time.

### Comparing SAILnet receptive fields to those observed in macaque V1

In many theoretical studies (*e.g.*, [1], [12], [22], [23]), one learns a sparse coding dictionary for natural stimuli, then compares the shapes of the resultant basis functions to the shapes of the physiologically measured receptive fields.

Typically, this comparison is either done by eye (as in [22]), or by fitting both the model RFs and the experimentally measured ones to some parameterized shape functions, and then comparing (again, typically by eye) the distributions of the resultant shape parameters for both the model and the data [1], [12], [23]. More rigorously, one can quantitatively compare the distributions of these shape parameters (Rehn, Warland, and Sommer, CoSyNe 2008 abstract) between the model and the experimental data, although that method fails if one has too few RFs with which to perform the comparison.

The by-eye comparisons are not very quantitative, even if they first involve fitting parameterized shape models, and any fitting of parameterized shape models is vulnerable to failures of the shape function: any RFs whose shapes are not well described by the parameterized function will yield nonsense best fit parameter values.

To get around these difficulties, we introduce a novel method for directly comparing the shapes of theoretical and experimental receptive fields, using image registration. In this technique, we assume that receptive fields may differ by a translation, rotation, and/or global size rescaling, yet still have the same shape. For example, consider an equilateral triangle within a bounding box. A shifted, rotated, and resized version of that shape is still an equilateral triangle.

We apply this intuition to the comparison between our model receptive fields, and a set of 250 macaque V1 receptive fields courtesy of D. Ringach. We do this by taking each experimentally measured V1 receptive field and then for each model RF we find the combination of translation, rotation, and overall rescaling that gives the best match between the experimental and transformed-model RF. We quantify the match by the 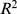 value (square of the correlation coefficient) between the pixel values of the model and the experimental RF. Choosing the 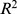-maximizing RF is similar to seeking the model RF that can account for the largest fraction of the variance of the experimental RF, but allows for an overall multiplicative constant in front of the model RF. Specifically, the 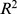 value tells us the fraction of the experimental RF variance that could be explained by a linear function of the best model RF (*i.e.*, possibly including an additive constant to all pixel values and an overall amplitude change).

Once we have done this for all model RFs, we take the one whose best transform yields the largest 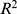 and take that as the best-fit model RF for the given experimental RF. In this way, we answer the question: Once we account for possible translations, rotations, and size rescalings, how much of the variance in the experimental RF pixel values can be accounted for by the library of model RFs? 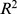 values near 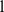 indicate that the experimental RF is reproduced perfectly by the theoretical model, while values near 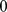 indicate that it is not. We then repeat this procedure for all of the RFs in the experimental dataset, yielding one 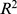 value per experimental RF. Below, we discuss the averages over these 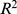 values.

**Figure 6:**
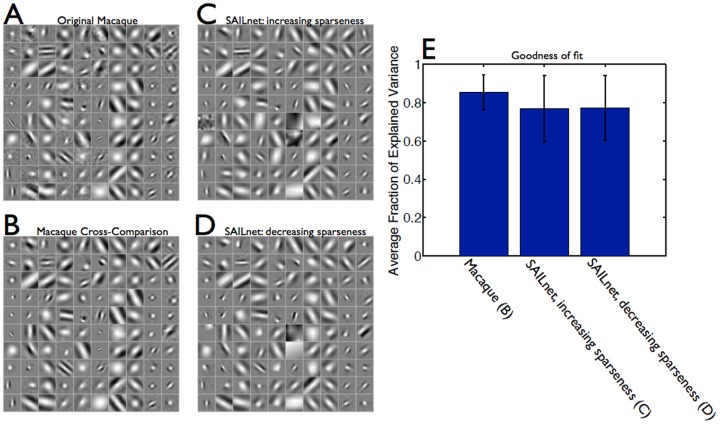
Registration-based receptive field comparisons show a quantitative match between SAILnet and macaque V1 RF shapes. (**A**) For illustration purposes, we show 100 of the 250 macaque V1 receptive fields (courtesy of D. Ringach) against which we compared our SAILnet model neuronal RFs. To estimate the fraction of the variance in the pixel values of these RFs that could realistically be explained, we performed registration-based RF fitting for each macaque RF, using *all other macaque RFs* as comparators. The best-fit matches to the RFs in panel **A** are shown in panel **B**, and the distribution of 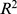 values obtained with the macaque-vs-macaque fitting shows that, on average, approximately 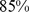 of the variance in the macaque RF pixel values can be explained by other macaque RFs (**E**). For the SAILnet model that experienced increasing sparseness during training, the best-fit RF matches are shown in panel **C**, and the distribution of corresponding 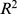 values shows that, on average, approximately 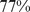 of the variance in the macaque RFs can be explained by these SAILnet model neuronal RFs (**E**). Similarly, for the SAILnet model that experienced decreasing sparseness during training (**D**), approximately 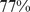 of the variance in the macaque RFs can be explained by model neuronal RFs. For both of the SAILnet networks shown (**C,D**), there are a few macaque RFs for which the image registration fails completely. We include these in our goodness-of-fit statistics; they correspond to the cells with 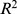 values near zero. There is no clear trend in the shapes of macaque RFs that cause this failure. For either increasing, or decreasing sparseness during training, the model neuronal RFs can, on average, account for approximately 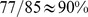 of the explainable variance in the measured macaque RFs. The error bars on the bars in panel (**E**) correspond to the standard deviation of the 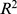 values over the sample of experimental RFs. There is a statistically significant, although small in magnitude, difference between the quality with which the macaque RFs fit each other (**E**), and the quality with which either model fits the data (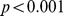 for either model, using a paired t test; n = 250). There is no statistically significant difference between the quality with which the two models fit the macaque RFs (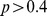, paired t test; n = 250).

Looking at the macaque RFs in Fig. 6A, it is clear that the region of support of the RF is often smaller than the size of the image window over which the RF is measured, and any noise outside of the region of support (or within it, for that matter) will be unaccounted for by the image registration process, thus lowering the apparent goodness-of-fit. To attempt to quantify this level of noise and to give a solid benchmark with which to assess our experiment-vs.-model comparison, we repeat the image registration fitting described above, but instead of using model RFs as comparators, we compare each experimental RF against the corpus of *other experimental RFs*. In other words, we do a leave-one-out analysis where we try to fit each macaque RF and, for that fit, we take the 249 other macaque RFs and pretend that they are “model” RFs. We then find the largest 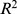 value possible for the best transformation of each of the 249 comparison RFs and use that number to quantify how well we could realistically expect to fit the macaque RF. We term this maximal 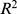 value for the macaque-to-macaque comparison the fraction of variance in the data that is “explainable” by our image-registration technique (although not necessarily captured by the dictionary of shapes learned by our sparse coding model).

In using this technique to estimate the noise level, we essentially assume that the RF shapes are repeated in the experimental data (which one can see in Fig. 6), and use that intuition to ask, “How much of the data variance is due to noise rather than the RF properties themselves?”

In Fig. 6, we show the experimental RFs, the best-transformed (translation, rotation, and overall size rescaling) model RFs learned with either increasing or decreasing sparseness values, and the quantitative comparisons between the RF shapes (average 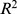 values for how well the macaque RFs can be explained by the model RFs).

The macaque-to-macaque comparisons (Fig. 6E) show that, on average, 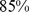 of the variance in the RF pixel values can be explained using other RFs from the macaque V1 dataset. We term this the “explainable” variance and it sets an upper bound on how well we could expect our model RFs to match the macaque RFs. For comparison, the model RFs account for, on average, 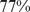 of the variance in the RF pixel values, regardless of whether the learning of those RFs was accompanied with either increasing or decreasing sparseness. The difference between the average explained variance for the two different models (those of Figs. 3 and 4) is not statistically significant (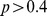, paired t test; 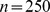), while the differences between each of the model's mean 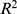 values and that of the macaque-vs-macaque comparison are statistically significant (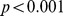, paired t test; 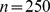).

Because the model RFs can explain an average of roughly 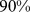 (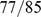) of the explainable variance in the RF data, regardless of whether the model experienced increasing or decreasing sparseness during training, we conclude that the model RFs are in quantitatively good agreement with the experimental RFs, independent of whether the learning of shapes was accompanied with increasing or decreasing sparseness.

Generally, larger networks have a greater diversity of receptive field shapes (this can be easily seen by comparing the RFs shown in this paper to those in [12]), and will thus tend to perform better in our image-registration comparisons. The networks studied in this paper were relatively small, in order to allow us to study the evolution of networks with many different initial conditions. Thus, we expect that even better model-to-experiment RF matches are possible if one were to study larger networks. On the other hand, the macaque V1 database to which we compared our model contains only 250 receptive fields, so a “fair” comparison to a larger simulated network would require more experimental data, or the selection of a random subset of the simulated RFs.

Our quantitative non-parametric RF comparison method could be used to compare many different theories to experimental data, and thus to ascertain which ones provide the best fit. That comparison is beyond the scope of this paper.

## Discussion

### Main contributions of this work

We have demonstrated that a computational model (SAILnet [12]) can learn V1-like receptive fields while simultaneously exhibiting either a *decrease*, or an *increase*, in the sparseness of neuronal activities. In both cases, the sparseness of the final (mature) network state is high enough to be reasonably considered “sparse.” We further showed that these same trends in sparseness over time can be achieved with the less biophysically realistic SparseNet model, despite the fact that it does not incorporate the same form of homeostasis as SAILnet.

In order to quantify the similarity between experimentally measured V1 receptive fields and the receptive fields learned by our SAILnet model, we have further introduced a novel non-parametric RF comparison tool based on image registration techniques.

Since sparseness can decrease during development, with the mature network state still performing sparse coding, the type of active sparseness maximization disproven by recent experiments [14] is not necessary to produce observed V1 receptive field shapes, nor is it required to learn a sparse representation of natural scenes. The trends in sparseness over time can be so strongly affected by the initial conditions of the network that those trends are not very informative about the objective function being optimized. Thus, developmental data, such as those shown in Fig. 1, cannot strictly rule out the general notion that V1 simple cell receptive fields develop so as to form sparse, efficient, representations of natural scenes.

### Is it fair to compare the sparseness trends experienced by developing animals with those exhibited by sparse coding models?

One possibility that requires consideration is that the sparseness data of Berkes and colleagues [14] should not be compared at all with theoretical models when assessing the hypothesis that V1 performs sparse coding. Recall that the sparse coding models criticized by Berkes and colleagues showed increasing sparseness *during receptive field formation*, and that their claim was that, since the ferret data instead showed decreasing sparseness *during development*, the sparse coding models do not provide a good description of V1.

In order for this comparison to be “fair,” one must ensure that receptive fields undergo significant change during the developmental period over which Berkes and colleagues measured sparseness. Indeed, other experimenters have observed that the orientation and direction selectivity of the neurons in ferret V1 increase [24], [25] during the same developmental period over which Berkes and colleagues observed decreasing sparseness levels, and that the mature state of the visual cortex for both ferrets and cats is sensitive to visual experiences in this period [25]–[28]. Combining these observations, we note that sparseness in ferret V1 seems to decrease while the visual cortical maps and receptive fields are being refined by experience, in apparent contradiction to the SC hypothesis. The largest contribution of this paper is to resolve that apparent contradiction.

Of course, there could always be other reasons — beyond the scope of this paper — why the developmental data fail to be relevant to the sparse coding hypothesis. We leave that question for future work.

For the sake of completeness, we note that Rochefort and colleagues have observed that *spontaneous* slow-wave activity in the anesthetized mouse visual cortex becomes sparser immediately after eye-opening [29]. At first glance, this might appear to contradict the ferret data of Berkes and colleagues [14]. However, since the ferret data is stimulus-evoked activity and the mouse data is spontaneous activity, it is not clear that a comparison between these datasets is meaningful. Because the spontaneous activity is not very easily related to the sparse coding hypothesis, which does not have much to say about activity in the absence of sensory input, we have focused on the ferret data (stimulus-evoked activity) in this paper.

### Prior work on homeostasis and learning

We are not the first to propose that homeostasis might underlay experience-dependent modification of the nervous system. Indeed, Marder and others have strongly and persuasively argued that neural systems might have a desired operating point such that when perturbed they use homeostatic mechanisms to return to that desired functional state [18], [19]. Moreover, Miller and others have shown that homeostatic activity regulation can facilitate learning in model-neuronal systems [30]–[32].

Finally, recent work by Perrinet [30] also used homeostatic activity regulation in learning sparse codes, so that model may also show either increasing or decreasing sparseness over time, for appropriately chosen initial conditions.

### Concluding remarks

We have demonstrated that the mature network state can perform sparse coding regardless of whether learning is accompanied by an increase or decrease in sparseness. At the same time, sparse coding is not the only principle that has been proposed in order to understand V1 function. Of particular interest in this regard is a recent study by Berkes and colleagues [15]. In that work, the authors showed that both stimulus-evoked and spontaneous (in the absence of any stimulus) activity in V1 became more similar as the animals aged, suggesting that V1 might be learning a Bayesian prior on the statistics of the environment. They further observed that part of this change in activity distributions came about due to increases in the correlations between V1 neurons with age. This observation is contrary to the redundancy reduction arguments often used to support efficient coding models, such as traditional sparse coding models and SAILnet. Thus, there is some evidence that sparse coding may not be the best theory of V1 function, and that others, such as that advanced in [15], may be better in some regards. Moreover, it is not clear how the sparse coding hypothesis could account for many of the properties of V1 complex cells despite its success for simple cells, and differences in the level of activity between awake and anesthetized V1 may pose additional challenges for sparse coding models. However, sparse coding models have successfully accounted for the shapes of V1 simple cell receptive fields, while the Bayesian-type optimality models have yet to do so. There is thus much room for further advances in our understanding of sensory coding, and measurements that can rule out theoretical models are key to that advancement. Our results show that the decrease in sparseness during development, which has been argued to rule out sparse coding in V1 [14], is not, on its own, sufficient to make that claim.

## Methods

### Sparseness measures used in this work

There are many different ways to measure sparseness. In this work, we follow the experimental study of Berkes and colleagues [14] and use the following three measures.

First, the “activity sparseness” (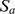), which is the fraction of units inactive in response to any given stimulus:
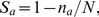
(1)where 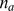 is the number of units whose activities rose above some threshold number of spikes in response to the stimulus, and N is the total number of units for which data were recorded. We set the threshold to 4 spikes for the multi-unit sparseness data shown herein. We performed this measurement by averaging over 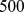 different input stimuli. The activity sparseness 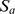 is very similar to the 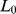 norm – which is actually a pseudo-norm – of the unit activities; low 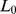 norms correspond to high 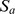 values.

In addition, we recorded two other sparseness measures, originally due to Treves 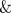 Rolls [20] (TR), and subsequently modified by Vinje 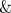 Gallant [5]. First, let us consider what we will call the “TR population sparseness” measure [15], 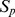,
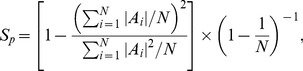
(2)where 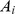 is the activity of unit 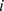. Note that 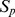 is assessed in response to a single image, although for our purposes, we will average this measure over 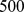 different image stimuli, to infer the average TR population sparseness. Similarly, we will define the “TR lifetime” sparseness of a given unit, 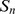, the same way (Eq. 2), but with the replacement that 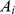 represents the unit's activity in response to a given image 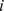, and 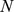 will be the number of different image stimuli (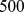) for which activities are recorded. Similar to the TR population sparseness 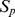, we will average these values over the entire population for our measurement.

### SAILnet model details

The SAILnet model [12] consists of a network of leaky integrate-and-fire neurons that receive feed-forward input from image pixels (a rough approximation of the thalamic input to V1), and inhibit each other through recurrent connections. The feed-forward weight from pixel 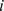 (with value 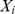) to neuron 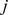 (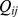) and the inhibitory recurrent connections between neurons 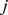 and 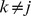 (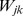) are learned by the network. In response to a given image, neuron 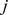 emits some number 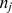 of spikes, which can be zero. Homeostasis, enforced via modifiable firing thresholds 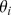 forces the neurons to all have the same lifetime-average firing rate of 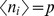 spikes per image. After the image presentation, the network parameters are updated via
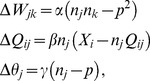
(3)where the (positive) constants 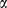, 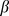 and 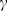 define the rates at which the parameters are learned. In order for them to remain inhibitory, after each update, the recurrent connections are rectified so that 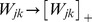.

### SAILnet simulation details

For all simulations shown herein, the feed-forward weights 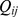 were initialized with Gaussian white noise, and the learning rates were set to 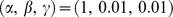. We used 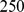 neurons which viewed 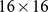 pixel image patches, hence the neuronal representation was approximately critically sampled (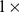 overcomplete) with respect to the number of pixels. The target firing rate was set to 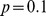 spikes per neuron per image in all cases. In all cases, the 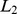 norm of the feed-forwards weights to each neuron was 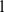 in the initial condition.

For the data shown in Fig. 4, the initial recurrent connection strengths 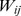 were drawn randomly from a normal (Gaussian) distribution with zero mean and unit variance, the firing thresholds 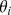 were initialized to 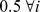, and the model neuronal activity became more sparse during training.

For the results shown in Fig. 3, the initial recurrent connection strengths were drawn randomly from a lognormal distribution (the negative of their logarithms were drawn from a normal distribution with zero mean and unit variance), the firing thresholds were drawn uniformly over 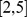, and the model neuronal activity became less sparse during training.

We note that in all cases, the specific shape of the sparseness vs. time plot depends on the choice of initial conditions. However, for a large class of initial conditions, the sparseness will decrease over time, and for another large class of initial conditions, it will increase (data not shown). Thus, our qualitative conclusion is not particularly sensitive to the exact numerical values described above.

### SparseNet simulation details

The SparseNet results were generated using code publicly distributed by Bruno Olshausen (http://redwood.berkeley.edu/bruno/sparsenet/). The code was used “out of the box”, without modifying the parameter values. For the data shown in Fig. 5a, then bases (matrix A in the code) was initialized with Gaussian white noise of variance 1. For the data shown in Fig. 5b, matrix A was initialized with Gaussian white noise of variance 0.01.

In both cases, 256 units were used, and the model was trained on 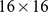 pixel image patches: the model is 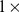 overcomplete with respect to the number of input pixels.

### Registration-based RF comparisons

To perform the quantitative comparison of receptive field shapes, we used the image registration tool in MatLab [33]. This package allowed us to quickly and easily compute the optimum combination of translation, rotation, and stretch – called a “similarity” transform in MatLab – to match each physiology RF with an RF from the appropriate comparison class (either model data or other experimentally measured RFs). For the optimizer, we used the “monomodal” option.

Our data consists of 250 Macaque V1 receptive fields, measured using reverse-correlation methods in the lab of Dario Ringach [34]. These data are either 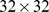, 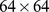, or 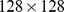 pixel images, showing the extent to which the neuron responds to each pixel. In order to standardize these data for the comparison, and because many of the RFs occupy only a tiny fraction of the image, we pre-process those RFs that are larger than 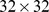 pixels, as follows. First, we find the peak absolute pixel value in the image – nominally, this is somewhere in the region of support of the “real” RF –, and we cut out a 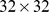 pixel region surrounding that peak. We then perform our image registration fitting on these standard-sized RFs.

For each macaque RF, we performed an exhaustive search over all model RFs, wherein we found the best similarity transform to match each model RF to the macaque RF, then took the best-matching model RF (with the appropriate best similarity transform), as the fit. The 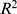 value between this best-fit transformed-model RF and the data RF was used to quantify the goodness of fit.

To generate a benchmark to assess how good a “good” 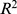 value is for this problem, we repeated our fitting process, but instead of fitting the data to SAILnet model RFs, we fit each macaque RF with the corpus of *other* macaque RFs. In so doing, we could estimate the fraction of data variance that could be explained in a best-case scenario.

The ratio between these numbers – the data-vs.-model 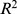 value and the data-vs.-data 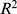 value – gives us an estimate of the fraction of explainable variance that is captured by the model.
